# Traumatic Left Renal Vein Thrombosis Associated With a Grade IV Renal Laceration: A Case Report

**DOI:** 10.7759/cureus.117685

**Published:** 2026-10-09

**Authors:** Youness Tahri, Anouar El Moudane, Youssef Kouiss, Mohammed Ramdani, Ali Barki

**Affiliations:** 1 Urology, Faculty of Medicine and Pharmacy of Oujda, Oujda, MAR; 2 Urology, Mohammed VI University Hospital Oujda, Oujda, MAR; 3 Urology, Mohammed I University Oujda Faculty of Medicine and Pharmacy, Oujda, MAR

**Keywords:** blunt abdominal trauma, nephrectomy, renal laceration, renal vein thrombosis, traumatic renovascular injury

## Abstract

Renal vein thrombosis (RVT), defined as the presence of a thrombus in the main renal vein or its tributaries, is a rare clinical entity. While traumatic RVT usually occurs in combination with severe arterial or parenchymal damage, isolated renal venous involvement without arterial injury is extremely rare. We report a case of left renal vein thrombosis following blunt abdominal trauma, without accompanying renal arterial damage. The diagnosis was established by contrast-enhanced computed tomography (CECT) and confirmed intraoperatively. Surgical options range from thrombectomy to nephrectomy. In our patient, kidney salvage was not feasible due to persistent hemodynamic instability and severe associated injuries, necessitating an emergency hemostatic nephrectomy.

## Introduction

Renal vein thrombosis (RVT) is commonly associated with conditions such as nephrotic syndrome, tumor thrombus, primary retroperitoneal disorders causing venous compression, oral contraceptive use, corticosteroid therapy, renal transplantation, trauma, and renal tumours. In blunt abdominal trauma, renovascular injuries are exceptionally rare, accounting for less than 1-2% of all renal traumas, with isolated venous involvement with complete renal artery preservation representing a minute fraction of these cases [1].

The underlying pathophysiology of RVT is governed by Virchow’s triad, which includes [2]: endothelial injury resulting from direct damage to the vessel wall; stasis of renal blood circulation; and a local or systemic hypercoagulable state.

The diagnosis can be promptly confirmed using contrast-enhanced computed tomography (CECT) imaging [3]. The overall incidence of RVT varies depending on the underlying etiology, with males being more frequently affected than females. Herein, we present a rare case of left renal vein thrombosis following blunt abdominal trauma without accompanying renal arterial injury.

## Case presentation

A 55-year-old male with an unremarkable medical history was admitted to the emergency department six hours following a high-energy road traffic accident involving a collision between his motorcycle and a truck. Upon arrival, the patient was hemodynamically unstable, presenting with a blood pressure of 68/44 mmHg and a heart rate of 110 beats per minute.

Initial clinical examination revealed left lumbar pain and tenderness, as well as tenderness in the left iliac fossa. There was no gross hematuria, lumbar ecchymosis, or open wounds on the abdomen or back. Examination of the external genitalia was unremarkable. Initial laboratory tests demonstrated a hemoglobin level of 7.9 g/dL, a platelet count of 221,000/µL, and a prothrombin time of 76%, with other biochemical parameters remaining within normal limits.

An emergency abdominal contrast-enhanced computed tomography (CECT) scan was performed, demonstrating a Grade IV left renal injury according to the American Association for the Surgery of Trauma (AAST) classification system, alongside a moderate left perirenal retroperitoneal hematoma (Figure 1).

**Figure 1:**
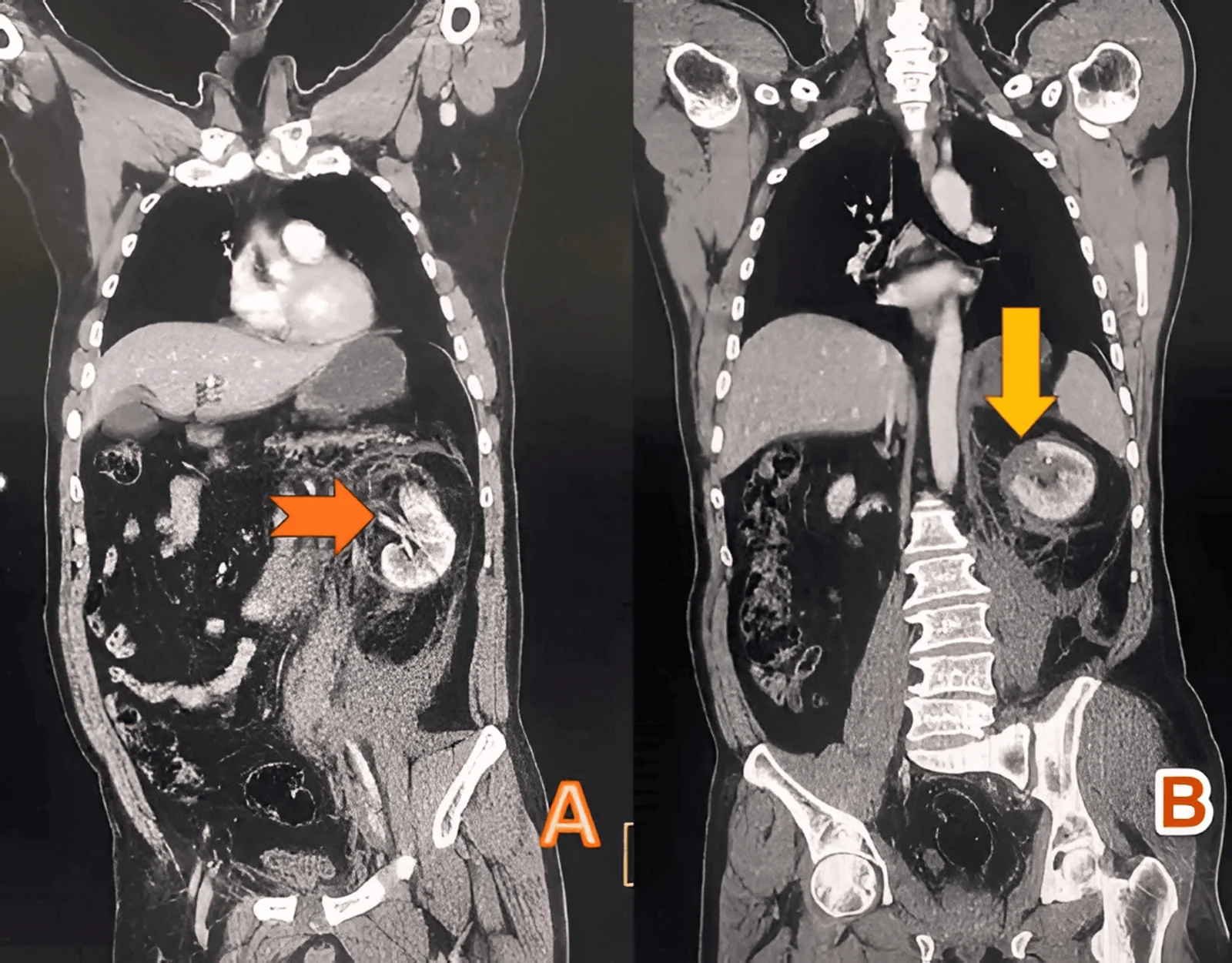
Abdominal CECT scan showing (A) preservation of the left renal arterial pedicle and (B) left upper pole renal parenchymal laceration with moderate retroperitoneal hematoma CECT: contrast-enhanced computed tomography

The CECT scan further revealed an irregular left renal vein wall with a 42-mm-long circumferential parietal hematoma extending to the hilar region, causing downstream renal vein thrombosis. Additionally, severe laceration of the upper pole renal parenchyma was noted, while the left renal artery remained fully patent (Figure 2).

**Figure 2:**
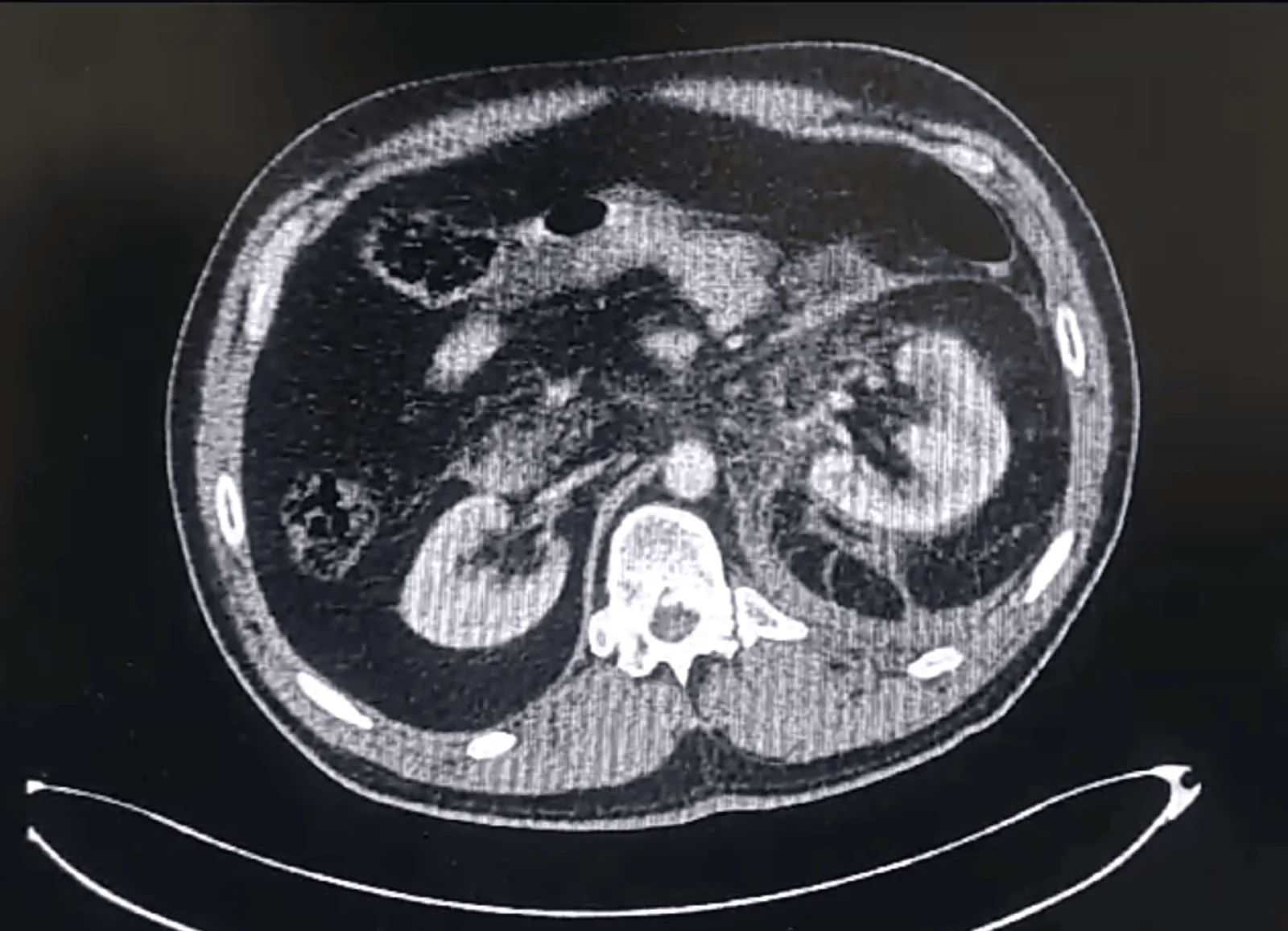
Abdominal CECT image demonstrating left renal vein parietal thickening and downstream renal vein thrombosis CECT: contrast-enhanced computed tomography

Due to persistent hemodynamic instability despite active fluid resuscitation, the patient was immediately taken for surgical exploration. Intraoperative findings confirmed a left upper pole renal parenchymal laceration associated with complete left renal vein thrombosis (Figure 3). A concomitant splenic rupture was also identified. An emergency hemostatic left nephrectomy and splenectomy were performed. The patient recovered well postoperatively and was discharged in stable condition.

**Figure 3:**
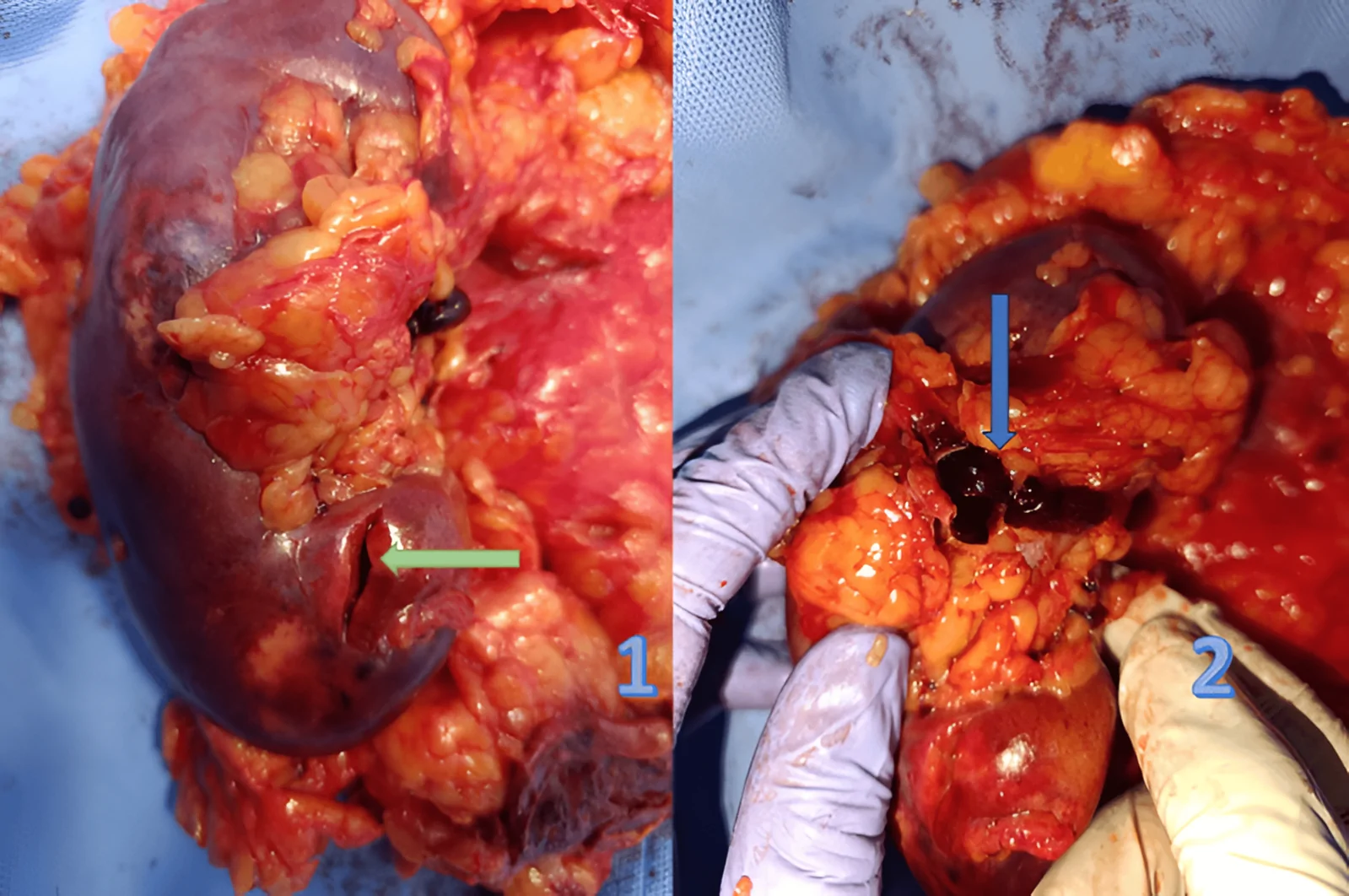
Intraoperative photographs demonstrating (1) left upper pole renal parenchymal laceration and (2) a thrombosed left renal vein

## Discussion

RVT, defined by the presence of a thrombus in the main renal veins or their tributaries, is a rare pathological entity in trauma, with a higher reported incidence in males and no known racial predilection [4]. In our patient, there was no prior history or clinical evidence of hypercoagulable states, nephrotic syndrome, or malignancy that could account for spontaneous thrombus formation.

In 1856, Rudolf Virchow delineated the pathophysiology of venous thrombosis through three interrelated factors: endothelial vessel damage, stasis of blood flow, and hypercoagulability-collectively known as Virchow’s triad [4]. In blunt abdominal trauma, violent deceleration forces exert severe traction on the renal pedicle, leading to intimal disruption, local stasis, and subsequent intravascular thrombosis.

Anatomically, the left renal vein is significantly longer than the right and lacks the mechanical posterior tethering offered by the inferior vena cava (IVC) to the right renal vessels [5]. This greater anatomical mobility predisposes the left renal vein to violent movement during abrupt deceleration, explaining the higher incidence of left-sided traumatic venous injuries. In contrast, the right renal vein is shorter and tethered by the IVC, which significantly restricts its movement and lowers the risk of traction injury [5]. However, the exact biomechanical mechanism that leads to isolated venous injury while completely sparing the adjacent renal artery remains rare and incompletely understood. Similar cases of traumatic renal vein thrombosis following blunt trauma have been described in the literature, emphasizing the role of localized parietal injury [6].

The classical clinical presentation of RVT includes flank pain and hematuria. When thrombus propagation extends into adjacent vascular structures, such as the gonadal vein or the IVC, it may present with testicular pain, lower extremity edema, or life-threatening pulmonary embolism [7]. Contrast-enhanced computed tomography (CECT) remains the gold-standard diagnostic tool to assess venous patency, renal perfusion, and associated intra-abdominal organ damage.

Management strategies for traumatic RVT range from conservative surveillance and anticoagulation/thrombectomy to nephrectomy [8]. In stable patients with isolated vascular lesions, conservative management or revascularization is preferred to preserve renal parenchyma, as early nephrectomy has been associated with an increased long-term risk of renal insufficiency [9]. However, early nephrectomy is indicated as a life-saving intervention in the setting of severe parenchymal destruction, complete devascularization, or refractory hemorrhagic shock [8,9]. In our patient, the combination of a Grade IV renal laceration, renal vein thrombosis, and concomitant splenic rupture caused persistent, life-threatening hemodynamic instability that failed to respond to fluid resuscitation, leaving emergency hemostatic nephrectomy and splenectomy as the necessary life-saving procedure.

## Conclusions

Traumatic renal vein thrombosis remains a rare and challenging diagnosis following blunt abdominal trauma. Physical examination and routine laboratory tests lack sensitivity for early detection, making immediate contrast-enhanced computed tomography (CECT) essential for timely evaluation. While organ-preserving strategies and conservative management are preferred in hemodynamically stable patients, emergency hemostatic nephrectomy remains a critical, life-saving intervention when severe vascular injuries are complicated by parenchymal destruction, associated visceral trauma, and refractory hemorrhagic shock.
